# Validation of a cerebral hemodynamic model with personalized calibration in patients with aneurysmal subarachnoid hemorrhage

**DOI:** 10.3389/fbioe.2022.1031600

**Published:** 2022-11-25

**Authors:** Yuanyuan Shen, J. Joep van der Harst, Yanji Wei, Reinoud P. H. Bokkers, J. Marc C. van Dijk, Maarten Uyttenboogaart

**Affiliations:** ^1^ Department of Neurosurgery, University Medical Center Groningen, University of Groningen, Groningen, Netherlands; ^2^ Department of Neurology, University Medical Center Groningen, University of Groningen, Groningen, Netherlands; ^3^ Eastern Institute for Advanced Study, Yongriver Institute of Technology, Ningbo, China; ^4^ Department of Radiology, Medical Imaging Center, University Medical Center Groningen, University of Groningen, Groningen, Netherlands

**Keywords:** cerebral hemodynamic, circle of Willis (CoW), numerical modeling, subarachnoid hemorrhage, transcranial Doppler (TCD)

## Abstract

This study aims to validate a numerical model developed for assessing personalized circle of Willis (CoW) hemodynamics under pathological conditions. Based on 66 computed tomography angiography images, investigations were obtained from 43 acute aneurysmal subarachnoid hemorrhage (aSAH) patients from a local neurovascular center. The mean flow velocity of each artery in the CoW measured using transcranial Doppler (TCD) and simulated by the numerical model was obtained for comparison. The intraclass correlation coefficient (ICC) over all cerebral arteries for TCD and the numerical model was 0.88 (N = 561; 95% CI 0.84–0.90). In a subgroup of patients who had developed delayed cerebral ischemia (DCI), the ICC had decreased to 0.72 but remained constant with respect to changes in blood pressure, Fisher grade, and location of ruptured aneurysm. Our numerical model showed good agreement with TCD in assessing the flow velocity in the CoW of patients with aSAH. In conclusion, the proposed model can satisfactorily reproduce the cerebral hemodynamics under aSAH conditions by personalizing the numerical model with TCD measurements.

**Clinical trial registration**: [http://www.trialregister.nl/], identifier [NL8114].

## Introduction

The circle of Willis (CoW) plays a pivotal role in the distribution of blood throughout the brain and in the development of cerebrovascular disease (CVD). Knowledge of the blood flow through the CoW is frequently used in clinical practice, for instance to evaluate hemodynamic impairment in patients with an internal carotid artery occlusion. Furthermore, mechanical forces such as wall shear stress in individual vascular segments of the CoW has been found to correlate with intracranial aneurysm development (Tanweer et al., 2014). Thus, studying the hemodynamics of the CoW helps us understand the pathology of CVD.

Computed tomographic and magnetic resonance angiography (CTA/MRA) and transcranial Doppler (TCD) are the most commonly used techniques in clinical practice to collect information regarding blood flow through the CoW. Both CTA and MRA can accurately depict blood flow through the CoW but offer only morphological information. MR technics may provide additional information on the flow velocity and direction of blood flow in the CoW, but these methods are time-consuming and expensive (Van Laar et al., 2006; Amin-Hanjani et al., 2015; Hartkamp et al., 2018). TCD is an alternative noninvasive bedside tool that can be used to assess the proximal cerebral blood flow (CBF) but is highly operator dependent and limited by patients’ bone window conditions (Lui et al., 2005; Lorenz et al., 2009).

Numerical models have been proposed as an alternative method for determining blood flow through the CoW. Previous studies have shown that these models can assess multiple hemodynamic parameters, such as flow velocity and pressure throughout the CoW. Multiple studies have already revealed that various hemodynamic parameters are associated with the development of intracranial aneurysm and carotid artery stenosis. However, their conclusions are all conceptual, and no specific threshold of abnormal hemodynamics has been drawn yet. Numerical models are not used in clinical practice due to their complexity and unproved accuracy. The existing validated models have been attested at a group average level (Reymond et al., 2009) or with a very small number of participants (Reymond et al., 2011; Groen et al., 2018), failing to meet the required sample size of an accuracy test study [50 cases for an expected ICC of 0.8 (de Vet et al., 2011)]. Therefore, with unknown prediction error, it is hard to draw a threshold of abnormal hemodynamics on the basis of a numerical model for any CVD development, e.g., the rupture of aneurysm and tendency of plaque formation in the carotid artery. The prediction error of a numerical model is essential before its personalized application in clinical research.

Moreover, many of the validation studies have been performed in healthy volunteers (Helthuis et al., 2020) or in a physiological state (Alastruey et al., 2007; Liu et al., 2015), while the cerebrovascular environment significantly changes under pathological conditions, especially after the onset of aneurysmal subarachnoid hemorrhage (aSAH), when cerebral vasospasm is prone to occur, the arterial diameter does not vary equally throughout the CoW and the spastic artery might show a beaded pattern. Changes of vascular compliance is another important issue. Elevated blood pressure after subarachnoid hemorrhage (SAH) may lead to an increase in vascular smooth muscle cell tone, which contributes to increased cerebrovascular resistance not only at the level of the CoW (Warnert et al., 2016) (relating to the setting of stiffness constant) but also at the level of smaller arterioles (relating to the setting of boundary conditions). Whether a numerical model can assess CBF under such complicated situations is worth investigating.

In this study, we aimed to validate the accuracy of an in-house–developed numerical model (Shen, et al., 2022) that simulates CBF in the CoW in the subacute phase after aSAH, in which vasospasm reaches a peak at day 6–8 after the rupture of intracranial aneurysm (Weir et al., 1978). Prospectively collected CTA and TCD data of aSAH patients were used in this study, covering the spasm peak period to the greatest extent, thus guaranteeing that the study sample is representative of extensive pathological CBF conditions.

## Methods

This study was approved by the institutional ethical review board of the University Medical Center Groningen. The objection register was verified before data collection, and patient consent was therefore not required. The protocol of this study has been previously published (Shen et al., 2020); the Netherlands Trial Register Identifier: NL8114.

### Subjects

Clinical and imaging data were collected from a previously completed prospective cohort study (the transcranial Doppler and CT angiography for investigating cerebral vasospasm in subarachnoid hemorrhage—TACTICS—study), which involved adult aSAH patients who had paired TCD and CTA performed at day 5 and day 10 after the onset of aSAH (van der Harst et al., 2019); TCD and CTA were performed within 24 h of each other.

### Clinical features

Demographics, blood pressure, Fisher scale, and clinical outcome information were obtained from the TACTICS database. High arterial blood pressure was defined as a systolic blood pressure (sBp) over 140 mmHg. The Fisher Grading Scale based on patients’ CT examination at admission was used to rate the severity of SAH (Fisher et al., 1980). Delayed cerebral ischemia (DCI) was defined as neurological deterioration and/or new ischemic lesions on follow-up imaging, not explained otherwise (van der Harst et al., 2019). The parent artery was defined as the artery with aneurysm, and the nonparent artery was the artery that was free from aneurysm.

### Transcranial Doppler

Flow velocity measurements with TCD were obtained by trained neurophysiology technicians, according to a standardized protocol (van der Harst et al., 2019). The mean flow velocity of nine cerebral arteries were collected: bilateral distal cervical part of the internal carotid artery (ICA) [C1 segment according to Bouthillier classification of ICA segments (Bouthillier et al., 1996)], bilateral proximal middle cerebral artery (MCA), bilateral anterior cerebral artery (ACA), basilar artery (BA), and bilateral posterior cerebral artery (PCA).

### CT angiography

CTA images were acquired on Siemens CT scanners (Siemens AG, München, Germany) in accordance with a standardized imaging protocol, and the CTA images were reconstructed to 0.60–0.75 mm slices (van der Harst et al., 2019). Two physicians (Y.S. and M.U.), blinded to the TCD results and clinical information, evaluated the images for scan quality and measured the diameter of the distal and proximal points of each artery from the brain CTA using the TeraRecon AquariusNET iNtuition Viewer [V.4.4.13 (P4)]. The CTA was excluded if the images were determined as uninterpretable due to movement, insufficient contrast in the arteries, or severe artifacts due to clip/coils.

**FIGURE 1:**
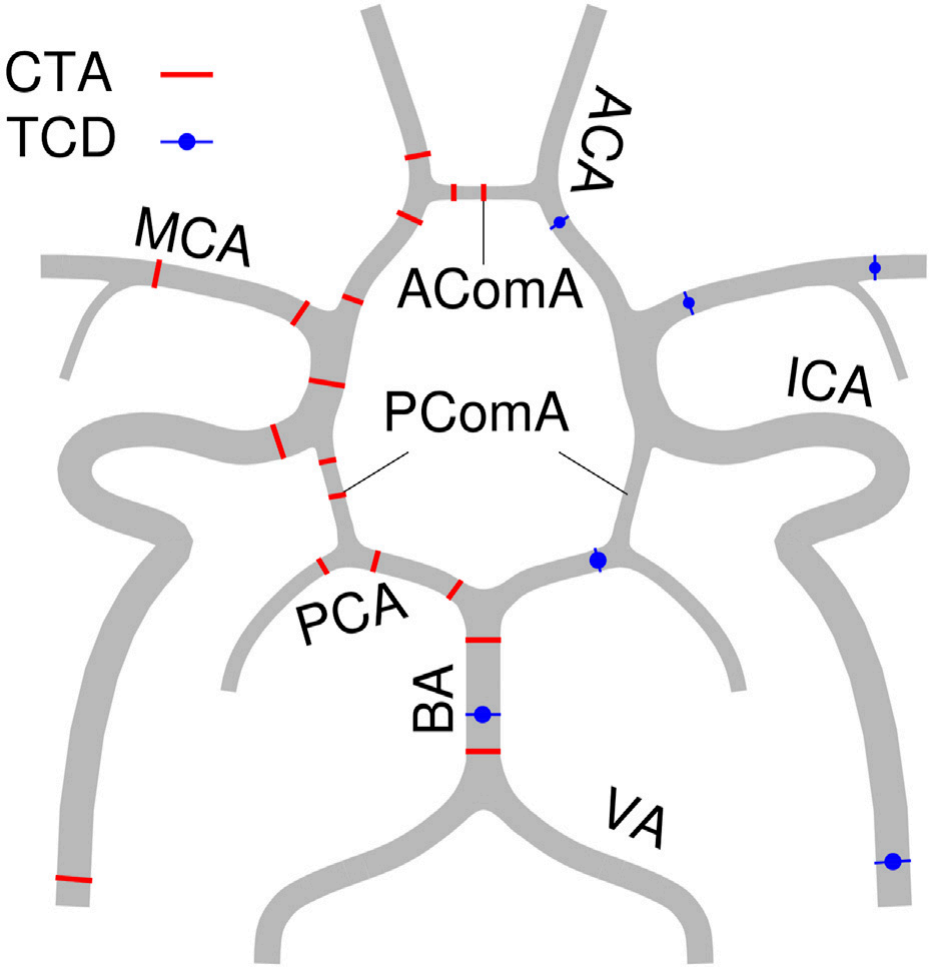
Locations of CTA and TCD measurements.

Three points of each ACA, MCA, PCA, and ICA, as well as two points of BA and three communicating arteries were measured for simulation. The details of these locations have previously been described in the study protocol (Shen et al., 2020) and is sketched in Figure 1.

In the numerical model, each artery segment is depicted as a uniform thin and homogeneous deformable tube, the diameter of which is the volume-equivalent diameter by multiple measure points from CTA.

### Numerical model

**FIGURE 2:**
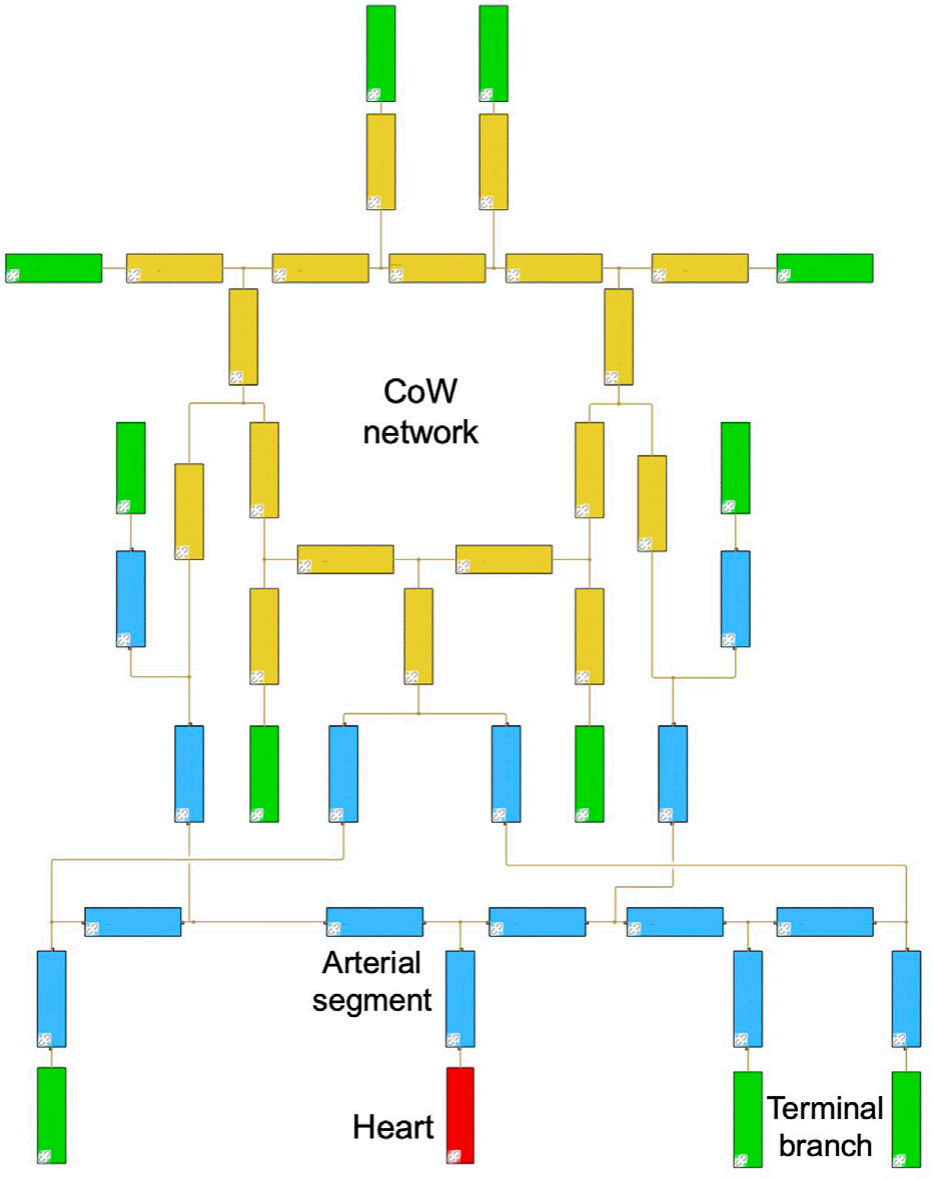
Block diagrams of the common arterial network.

The hemodynamic model used in this study is based on a block-diagram hydraulics toolbox, Simulink Simscape Fluids library. One of the distinguishing features of the present model is that it provides an intuitive and a simple way to construct patient-specific models by interconnecting a series of hydraulic components that represent artery segments. The applied common artery network consisted of 18 intracranial artery segments to capture flow feature in the CoW and 15 artery segments from the main body to offer a background blood flow. For personalized simulation, the input diameters were updated with the measured diameters per case. The diameters were assigned 0 if the artery segment was absent in CTA. By this way, the personalized CoW simulation was carried out with the common network as shown in Figure 2.

In the numerical model, the prescribed flow rate was applied at the root of the ascending aorta, and the three-element Windkessel model (WK3) was employed at each terminal branch to avoid unexpected reflection. The conventional approach (Alastruey et al., 2007) assumed that the flow rate was proportional to the cross-section area, as the peripheral resistance of each terminal branch was determined by the given flow distribution. This assumption may be invalid for patients with aSAH. A recent study (Helthuis et al., 2020) employed a structured tree and a simple autoregulatory model at the distal boundary conditions based on the measurement from healthy volunteers. It is unclear what the impact of aSAH is on edema and autoregulation, particularly during severe vascular spasms. The present numerical model proposed a three-step procedure, with the patient’s TCD and blood pressure measurements as input, to calibrate the personalized resistance and compliance of WK3. This procedure tailored the boundary of the Simulink model per patient per case under different aSAH conditions. The implementation of the three-step procedure is as follows:

1) Determining the peripheral resistance at each terminal branch with a linear electric circuit model, using the mean blood pressure and mean flow rate as input;
2) Correcting the Young’s modulus and peripheral compliance of the main body artery segments by using systolic and diastolic blood pressure as input;
3) Correcting the compliance of the cerebral arterial segments by adjusting the Young’s modulus of the cerebral segments based on the results of the tentative simulation in (2), using the systolic and diastolic flow rates as input.

This calibration procedure is important because peripheral resistance plays a dominant role in the flow distribution in terminal branches and segments in the CoW, and we found that the optimal peripheral resistance was highly scattered for patients with aSAH, as showed in Figure 5 in (Shen et al., 2020; Shen, et al., 2022).

The simulation ran for 10 cardiac cycles to achieve the steady periodic state that commonly takes 1 minute of computational time per case. Furthermore, the details of this numerical model have been described in previous studies (Shen et al., 2020; Shen, et al., 2022).

### Statistical analysis

The agreement on the mean flow velocity through the CoW from TCD and the numerical model was calculated by the intraclass correlation coefficient (ICC), based on a single rater type, consistency, and a two-way random model (Koo and Li, 2016). The Bland–Altman plot was generated to describe the correlation between the difference and mean of flow velocity measurements. Base 10 logarithmic transformations were performed to study the limits of agreement by percentile. High blood pressure, a high Fisher score (scale 3 or 4), close to ruptured aneurysm, and DCI were taken as potential negative factors associated with the accuracy of the numerical model. The ICC of each subgroup (dichotomized by blood pressure, Fisher score, location of ruptured aneurysm, and DCI) was considered to assess whether the prementioned factors were application restrictions of the numerical model. Statistical analyses were performed using SPSS version 25.0 (SPSS Inc.) and R version 4.0.2.

### Patient and public involvement

Data were collected from a completed prospective study. The objection register was checked before data collection. Informed consent was not required. The patients and public were not involved in the study design.

## Results

### Patient characteristics

Fifty-nine consecutive patients (37 women, 22 men, 57 ± 11 years) were included from the TACTICS database. Fifty-two investigations were excluded based on poor CTA image quality or long interval time between TCD and CTA. As such, 66 CTA data sets from 43 patients were available with sufficient image quality (Table 1). The study flow chart is presented in the supplementary 1.

**TABLE 1:** Baseline patient characteristics.

	Patients	Investigations
Total	43	66
Age (years)^a^	58.35 (10.35)	59.55 (10.21)
Sex^b^		
Female	31 (72.1)	48 (72.7)
Male	12 (27.9)	18 (27.3)
Fisher scale^b^		
1	1 (2.3)	2 (3)
2	3 (7.0)	4 (6.1)
3	19 (44.2)	30 (45.5)
4	20 (46.5)	30 (45.5)
Delayed cerebral ischemia^b^		
Present	7 (16.3)	7 (10.6)
Absent	36 (83.7)	59 (89.4)
Aneurysm location^b^ ^,^ ^c^		
Anterior circulation	31 (70.5)	49 (72.1)
AComA	17	26
M1	8	13
M2	3	6
A1	1	1
A2–3	2	3
Posterior circulation	13 (29.5)	19 (27.9)
P1	5	8
PComA	5	7
BA	3	4

AComA: anterior communicating artery; A1: pre communicating segment of anterior cerebral artery (ACA); A2: ACA, post communicating segment; A3: ACA, precallosal segment; M1: horizontal segment of middle cerebral artery (MCA); M2: MCA, insular segment; P1 pre communicating segment of posterior cerebral artery; PComA: posterior communicating artery; BA: basilar artery.

^a^ Mean (standard deviation);

^b^Case number (%).

^c^One patient had ruptured aneurysm on RM1 and unruptured one on LM1.

### Correlation between transcranial Doppler and numerical model

**FIGURE 3:**
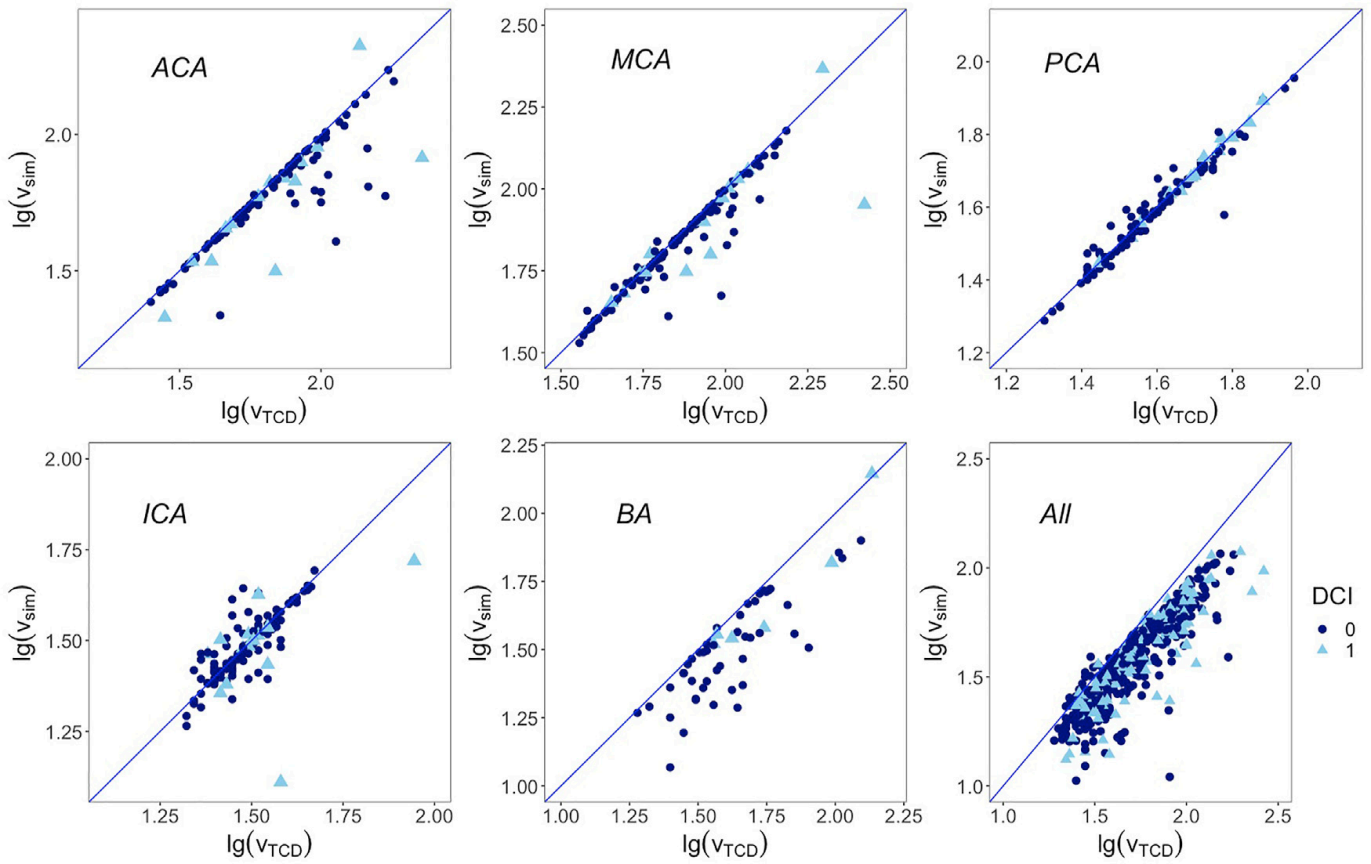
Correlation between base 10 logarithmic mean flow velocity from TCD on the x-axis and from simulation on the y-axis. Blue line represents the regression line y = x. First row from left to right: anterior cerebral artery, middle cerebral artery, and posterior cerebral artery; second row from left to right: internal carotid artery, basilar artery, and overall arteries. DCI: delayed cerebral ischemia. 1 represents cases that developed DCI afterward, 0 represents cases that are free from DCI.

TCD-measured mean flow velocities, paired difference between TCD and the numerical model, limits of agreement of the two methods, and ICC consistency are presented in Table 2. The ICC over all 568 arteries was 0.88 (95% CI, 0.86–0.90). The ICA had the lowest ICC of 0.75 (95% CI, 0.66–0.82), followed by ACA with 0.78 (95% CI, 0.70–0.84); the ICC of MCA, BA, and PCA was 0.85, 0.87, and 0.98, respectively. The scatter plots of the two logarithmic measurements grouped by DCI are presented in Figure 3.

**TABLE 2:** Agreement analysis per cerebral artery.

	Flow velocity	Paired difference^a^	Limits-of-agreement (%)	ICC (95%CI)	N	
Overall	-	-	-	0.88 (0.86 0.90)	568	
ICA	30.0 (27.0,35.0)	0.4 (−0.2, 1.0)	0.75, 1.36	0.75 (0.66, 0.82)	131	
MCA	69.5 (55.0,96.0)	1.4 (0.9, 3.1)	0.80, 1.39	0.85 (0.79, 0.89)	130	
ACA	67.0 (46.0, 85.0)	1.5 (0.8, 4.3)	0.73, 1.67	0.78 (0.70, 0.84)	127	
PCA	39.0 (30.0, 50.5)	0.8 (−0.3, 1.3)	0.89, 1.15	0.98 (0.97, 0.98)	125	
BA	38.0 (32.0, 53.0)	7.2 (2.1, 16.4)	0.81, 2.09	0.87 (0.78, 0.92)	55	

The mean flow velocity of each artery measured by TCD and paired difference from simulated flow velocity (V_TCD_, Vsimulated) were non-parametrically distributed. They are reported by median (interquartile range) in the unit cm/s.

Limits of agreement: in 95% cases, the simulated flow velocity may differ from TCD measurement by (1 − x) below to (x − 1) above.

ICC: intraclass correlation coefficient, single rater type, consistency, two-way random model.

^a^ The paired differences were not equal to 0 by Wilcoxon signed-rank test.

**FIGURE 4:**
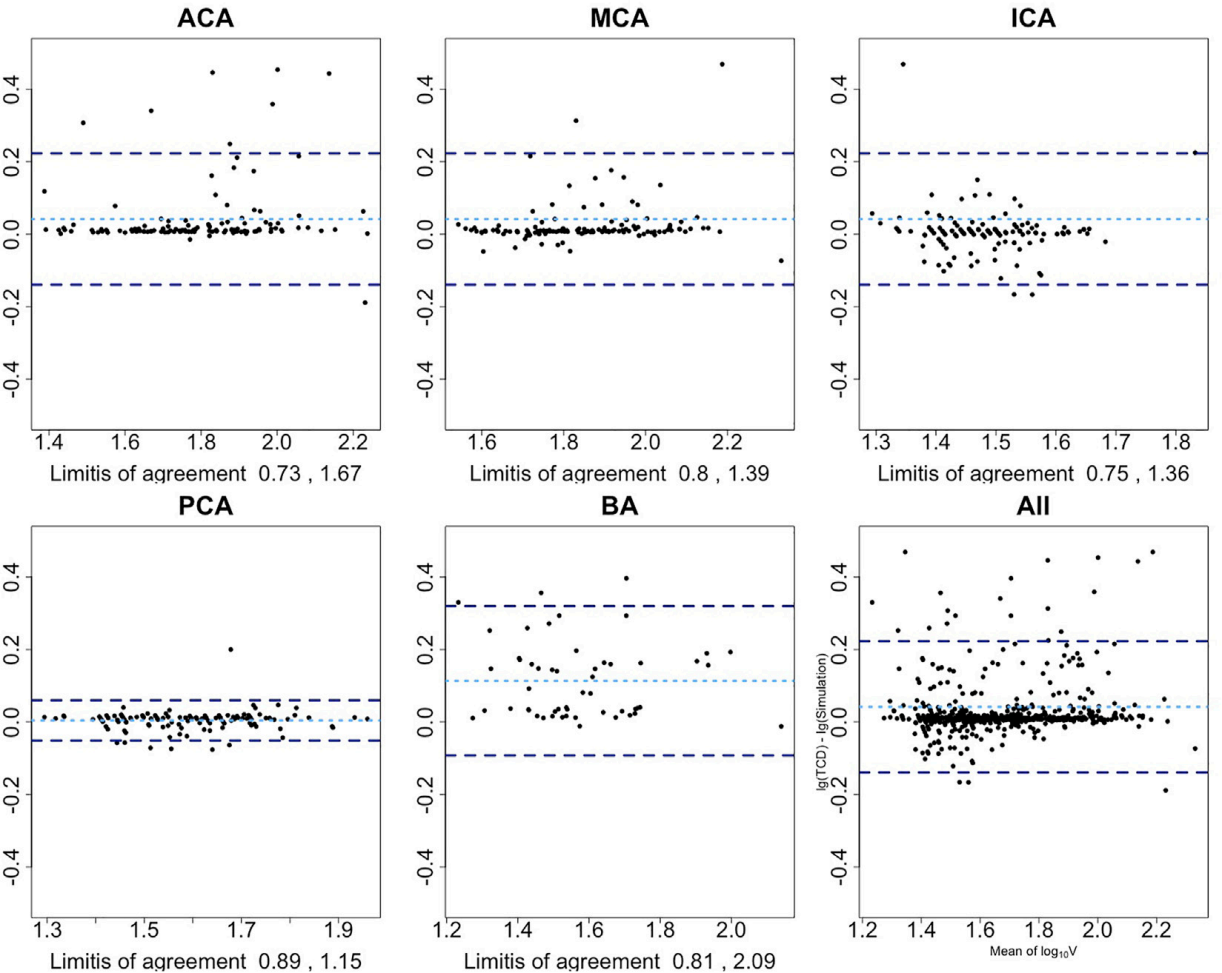
Bland–Altman plots show the agreement among the presented numerical model and TCD after base 10 logarithmic transformation. x-axis is the average mean of base 10 logarithmic flow velocity measured by TCD and simulation; y-axis is the difference between two logarithmic measures. Light blue dotted line represents the mean difference of two logarithmic velocities; two dark blue dotted lines represent 1.96 SD above or below the mean difference. Limits-of-agreement are listed at the bottom of each artery plot. First row from left to right: anterior cerebral artery, middle cerebral artery, and internal carotid artery; second row from left to right: posterior cerebral artery, basilar artery, and overall arteries.

The magnitudes of differences remained the same over the whole range of mean measurements in the Bland–Altman plots, except for the ACA and BA. Therefore, base 10 logarithmic transformations of the flow velocity were performed to yield logarithmic Bland–Altman plots, and the percentile differences are shown in Figure 4 (Martin Bland and Altman, 1986). The plots show a slight systematic underestimation of the numerical model when compared to TCD. The percentile differences are equally spread along the x-axis. The smallest antilogarithmic limits of agreements were in the PCA between 0.89 and 1.15, followed by the MCA (0.80 and 1.39), ICA (0.75 and 1.36), and ACA (0.73 and 1.67), while the widest were in the BA between 0.81 and 2.09.

**FIGURE 5:**
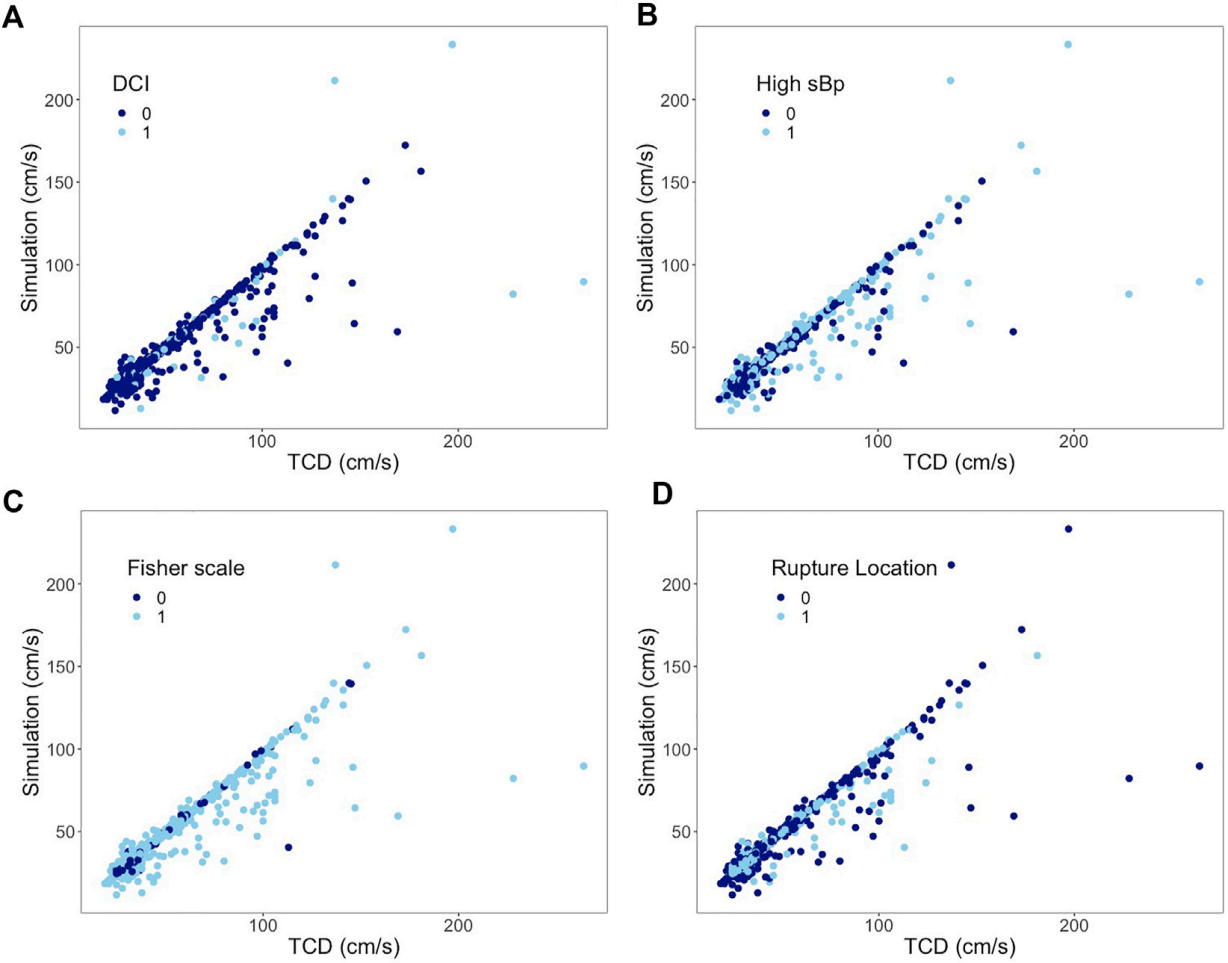
Subgroup analysis of all arteries. Correlation between mean flow velocities from TCD measurement on the x-axis and from simulation on the y-axis. **(A)** 0 represents non-DCI; 1 represents DCI. **(B)** 0 represents sBp ≤ 140 mmHg; 1 represents sBp higher than 140 mmHg. **(C)** 0 represents Fisher scale grade 1 or 2; 1 represents Fisher scale grade 3 or 4. **(D)** 0 represents ruptured aneurysm located at anterior CoW; 1 represents ruptured aneurysm at posterior CoW.

In the subgroup analysis, the ICC of the DCI group decreased by 0.2 when compared to the non-DCI group (ICC 0.93) without a 95% CI overlap of their ICCs. The ICC difference within the blood pressure, Fisher scale, and ruptured intracerebral aneurysm location groups was 0.04, 0.06, and 0.07, respectively; all overlapped within each pair’s 95% CI of the ICC (Table 3). The scatter plots of the paired subgroups are displayed in Figure 5.

**TABLE 3:** Subgroup analysis.

—	N	ICC (95%CI)	Limits-of-agreement (%)
Systolic blood pressure			
>140 mmHg	350	0.87 (0.85–0.90)	0.74, 1.53
≤140 mmHg	218	0.91 (0.88–0.93)	0.78, 1.46
Fisher scale			
3,4	515	0.88 (0.86–0.90)	0.75, 1.52
1,2	53	0.94 (0.90–0.97)	0.77, 1.41
Ruptured aneurysm location			
Anterior circulation	398	0.86 (0.83–0.89)	0.74, 1.53
Posterior circulation	170	0.93 (0.88–0.95)	0.77, 1.47
Delayed cerebral ischemia			
Yes	62	0.73 (0.59–0.83)	0.64, 1.92
No	506	0.93 (0.92–0.94)	0.77, 1.45

ICC: intraclass correlation coefficient, single rater type, consistency, two-way random model.

The ICC in the nonparent artery group was 0.87 (95% CI, 0.83–0.89), similar to the group with the parent artery (supplementary 2). The ICC in a sub–data set that excluded the second scan from 23 patients who offered two investigations is 0.87 (95% CI 0.83–0.90), similar to that of the full data set (supplementary 3).

## Discussion

This study validates our in-house–developed numerical model for simulating CBF in the CoW in patients with aSAH. With an ICC of 0.88 (N = 568; 95% CI 0.86–0.90), the model accurately simulated flow velocities, according to the ICC reporting guideline (poor if <0.5, moderate if between 0.5 and 0.75, good if between 0.75 and 0.9, and excellent if >0.9) (Koo and Li, 2016).

MRI is another noninvasive technique to measure the CBF. Previous studies have shown that the correlation between the MR technique and TCD is between 0.49 and 0.80 (Sorond et al., 2010; Meckel et al., 2013). Helthuis et al. (2020) validated a linear model with NOVA-MRI on simulating CBF among 20 healthy people and reported an ICC of 0.86 with the exclusion of 8 outliers out of 279 cerebral artery segments.

TCD is the most widely used application in clinical practice to determine CBF. However, it is not a perfect gold standard for cerebral flow velocity measurement. It is a highly operator-dependent instrument. The reliability of TCD in measuring CBF at the MCA has been reported to be between 0.76 and 0.82 depending on the patient’s bone window condition (Lorenz et al., 2009; Muñoz Venturelli et al., 2017). The reliability of the TCD measurements in the ACA and PCA may even be lower than those in the MCA. The expected validity of the numerical model, using TCD as a comparison test, cannot be more than the square root of the product of its reliability and the reliability of TCD. Therefore, the accuracy of the presented numerical model on simulating flow velocity has a maximum of 0.91 (Birnbaum et al., 1968). As such, the presented numerical model showed a good accuracy under various aSAH conditions over all cerebral artery segments measured in the CoW.

Scrutinizing the agreement at each cerebral artery, the presented numerical model showed a good agreement in the PCA, MCA, and ICA, with relatively bigger limits of agreement at the BA and ACA. In 95% of the cases, the simulated PCA flow velocity may differ from the TCD measurement by 11% below to 15% above; for the MCA, it is 20% below to 39% above, and 25% below to 36% above for the ICA.

The base 10 logarithmic Bland–Altman plot of the ACA reported a relatively wide range of limits of agreement with TCD from 27% below to 67% above. Its ICC also led to a relatively lower accuracy. When checking these outliers in Figure 3, it has been revealed that they belonged to patients who had developed DCI afterward. Whether the fast flow velocity or DCI caused a less accurate simulation in the ACA cannot be determined using the current data set. The other reason from the wide range of limits of agreement is that the ACA is divided into the A1 segment as the pre communicating part and A2 segment as the post communicating part in our numerical model, while in the TCD report only the flow velocity of the ACA was recorded, without a clear distinction between the A1 and A2 segments.

In 95% cases, the simulated BA flow velocity differed from the TCD measurement by 19% below to 109% above. The board range of limits of agreement is consistent with the range of its 95% CIs of ICC. This might be due to the setting of the BA in our numerical model. Though there are many paired arteries along the way from the beginning to the end of the BA (e.g., superior cerebellar artery), they are neglected in this lumped model. Such simplification shall lead to differences from reality. The other reason for this wide measurement error is insufficient data power for the BA analysis as we have reported in the study protocol (Shen et al., 2020). Therefore, the inherent measurement error of the BA segment in this numerical model might be overestimated with the current data set.

To understand the restrictions of the numerical model under various acute pathologic conditions, we stratified the data set by blood pressure, Fisher scale, location of ruptured aneurysm, and DCI. One may hypothesize that vasospasm might influence the accuracy of the numerical model under aSAH conditions. Vasospasm at the level of the CoW is correlated with the change of vascular smooth muscle cell tone, which would influence arterial wall stiffness in the numerical model. With vasospasm, arteries not only have reduced diameter but occasionally also exhibit a beaded pattern in their geometry. The influence of such a pattern is not accounted for in the presented model. The degree of local vasospasm is closely related to the amount of SAH and location of the ruptured aneurysm. Global and local edema after aSAH theoretically have sophisticated impacts on the setting of the numerical model as well.

The 95% CIs of ICC reported in Table 3 overlap between the paired subgroups stratified by the Fisher score or location of ruptured aneurysm, which means that the difference of paired ICC is random rather than a true difference. It implies that the model could simulate the flow velocity accurately regardless of the amount of SAH and bleeding location. High blood pressure was, furthermore, not found to affect the ICC between both techniques.

In the DCI group, the ICC was found to be lower without an overlap of their 95% CIs. Most of the outliners presented in Figure 3 are from patients who had developed DCI afterward. DCI as a complication might present during the course of aSAH, in which insufficient blood flow plays a conspicuous role (van Gijn et al., 2007). The boundary condition of the presented model, i.e., resistance, was calculated based on the Hagen–Poiseuille flow, which likely predominated the CBF of each terminal branch. The obtained resistance might have difficulties to properly derive the perfusion of each terminal branch under extreme pathological conditions which would lead to discrepancy. Whether a poor simulation by this model could identify DCI at the early course of aSAH is worth investigating in the future.

### Strength and limitation

This study is the first to validate a numerical model with sufficient population sampling and under the pathological condition of aSAH between day 5 and 10 after the onset. In order to present the model’s validity under all circumstances of aSAH and to explore the limitation of this numerical model, we included every sample after checking the outlier was not due to typing error (none was found). The simulation accuracy of each artery in the CoW is particularly reported by the measurement error, in favor of the presented numerical model’s personalized application for further clinical research. Its application limitations were further explored by multiple clinical parameters.

One limitation of the study design is the time interval between the artery morphology exam and flow velocity measurement. The aSAH patient is prone to suffer cerebral vasospasm which subsides by day 12 (Weir et al., 1978). Cerebral vasospasm after the onset is progressive and varies among individuals. Morphological information and flow velocity collected synchronously are thus important conditions for this validation purpose. MR technic can collect angiographic images and flow velocities simultaneously. However, due to the relatively long exam duration, severe patients (e.g., with delirium) are unable to endure the MR exam, which would lead to selection bias. An ideal study plan is thus impossible in practice for this research purpose. We excluded investigations where paired CTA and TCD performed longer than 24 h to avoid morphological changes, thus the diameter presented in the CTA image could represent the one while TCD was performed to the most extent.

Fifty-two investigations out of 118 were excluded due to the long time interval or poor CTA image quality. Besides the abovementioned reason, most investigations those were excluded were owing to severe image artifacts. All CTA images used in this study were captured after the repair of aneurysms. Thus, it is inevitable to have severe artifacts in some images that interfered with the segmental diameter measurement in the CoW. However, this exclusion rate might lead to selection bias.

Another limitation in this validation study has been the diameter measurements. As previously stated, the validity of the presented numerical model in this study setting highly depended on both the reliability of TCD and the numerical model. Since this numerical model took artery diameters as the input, its reliability was highly determined by the reliability of the measurements. To avoid bias, we used the semiautomatic central lumen calculation. The diameter measurement bias underestimated the ICC of the presented numerical model. Researchers using an automatic diameter-measuring tool can expect a more accurate CBF with this model. For others without a special image processing tool, the precision of the diameters measured from the daily image viewer can still accurately simulate the presented numerical model. Thus, the measurement bias could be the point of strength for external validation of this study.

The abovementioned limitations lead to another strength of this numerical model: it is applicable even for ordinary clinical practice without special equipment settings, while CTA and TCD are both common in a neurovascular center. Besides, based on arterial imaging stored in the medical files, the presented numerical model can even be applied in retrospective studies.

In addition, this study is an internal validation study, which tends to overestimate. An external validation should be carried out in the future.

### Clinical implications

When compared to the correlations of MRI techniques with TCD, mathematic models, and MR techniques for detecting CBF, the presented numerical model shows a good agreement even in the subacute phase of patients with aSAH. TCD, MR techniques, and the numerical model can detect patients’ CBF specifically and noninvasively. However, none of the three methods can measure the real *in vivo* blood flow. This study has shown that personalized hemodynamics of CBF simulation by our numerical model can act as a supplement to TCD and MR techniques by providing global hemodynamics of the CoW.

The present numerical framework integrated patients' commonly available morphology information and flow measurements to provide a comprehensive cerebral flow information. Time consumption of this numerical model from diameter measurement to personalized simulation on a PC was done within 20 min. The present study sheds light on the potential application of the hemodynamic model for clinical practices.

With personalized WK3 parameters, the model can assess the blood flow in the three communicating arteries, which are hardly detected by either TCD or MR exam, and the limited data measured can provide a global cerebral hemodynamic map that could serve as the boundary condition for higher level simulations, such as the 3D CFD model; it may be potentially used to investigate the hemodynamics of the CoW and CVD—e.g., a recent systematic review has demonstrated that despite the well-known association between the configuration of the CoW and the presence or rupture of intracranial aneurysm, a qualified hemodynamic assessment tool to identify such factors in clinical practices is still lacking (Shen, et al., 2022). The model may also be used to predict cerebral hemodynamic improvement if patients' CoW configuration get altered (e.g., during intracranial bypass operation and carotid endarterectomy).

## Conclusion

The presented numerical model can be a useful and generalizable tool for reconstructing personalized CoW hemodynamics by integrating limited clinical measurements. With this model, hemodynamic studies focused on CVD can be performed without special equipment setting.

## Data Availability

The raw data supporting the conclusions of this article will be made available by the authors, without undue reservation.
