# Supplementary material for: Validation of a cerebral hemodynamic model with personalized calibration in patients with aneurysmal subarachnoid hemorrhage
Source: Front Bioeng Biotechnol. 2022 Nov 25;10:1031600. doi: 10.3389/fbioe.2022.1031600 (PMC9732662; doi:10.3389/fbioe.2022.1031600)
Supplement: Supplementary file 1 [file Table1.PDF]

Supplementary 2

Subgroup analysis for parent artery

	ICC (95% CI)	N
**Overall**	0.89 (0.84-0.90)	568
**Parent artery***	0.96 (0.91-0.98)	26
**Non-parent artery**	0.87 (0.83-0.89)	542

*ACA:1, BA: 4, PCA:8, MCA:13. Aneurysm located at Acom, Pcom, M2 segment, and A2-3 segment were excluded from this group.

Parent artery: artery with the aneurysm; Non-parent artery: artery free from aneurysm.

ICC: intraclass correlation coefficient, based on single rater type, absolute agreement, two-way random model.

Comparing to ICC of all arteries, non-parent artery group had a similar ICC. The parent artery group had even higher ICC. It is because the parent arteries in our cases were mostly from PCA and MCA, which statistically showed higher ICC (see Table 1 and Table 2).

The presence of an aneurysm may significantly affect the local hemodynamics, e.g., resulting in local turbulent flow, abnormal wall shear stress, as reported in many 3D computational fluid dynamics (CFD) studies. The present study applied a lumped model which can describe the global hemodynamics on the arterial network but is incapable to capture the local hemodynamics. Hence, the presence of aneurysm was not taken into account in our numerical model.
