# Supplementary material for: Validation of a cerebral hemodynamic model with personalized calibration in patients with aneurysmal subarachnoid hemorrhage
Source: Front Bioeng Biotechnol. 2022 Nov 25;10:1031600. doi: 10.3389/fbioe.2022.1031600 (PMC9732662; doi:10.3389/fbioe.2022.1031600)
Supplement: Supplementary file 2 [file Table2.PDF]

Supplementary 3

Hierarchy data bias analysis

	ICC (95% CI)	N
**Overall**	0.89 (0.84-0.90)	568
**First scan**	0.94 (0.91-0.95)	262
**Second scan**	0.84 (0.79-0.87)	306
**Single set**	0.87 (0.83-0.90)	361

In Overall group all investigations were mixed for analysis; **First scan** group included investigations from day 5 after onset; **Second scan** group included investigations from day 10 after onset. **Single set** group excluded second scan set from 23 patients who offered two investigations to the dataset.

The above table shows no major differences in ICC between those subgroups, except first scan. The higher ICC in first scan group compare to that in second scan group might be explained by DCI cases. There were 7 patients developed DCI afterwards, they all offered only one investigation in the dataset: one was in the first scan group, six were in the second scan group. As we discussed in the paper, the numerical model simulates less accurate in the DCI group than non-DCI group. Thus, we concluded that no relevant bias had occurred due to the intra-individual correlation.

Another argument that justifies our methods to mix two investigations from the same patient, is that within a single patient, the hemodynamics and CoW configuration might have changed between the two investigations. aSAH patient is prone to suffer cerebral vasospasm which reaches a peak at day 6-8, subsides till day 12. Cerebral vasospasm after onset is progressive and varies between individuals.[1] This could be the reason of ignorable correlation between two investigations from the same patient in this study setting.

[1] B. Weir, M. Grace, D. Ph, J. Hansen, M. S, and C. Rothberg, "Time course of vasospasm in man," *J. Neurosurg.*, vol. 48, no. 2, pp. 173–178, 1978
