# Supplementary figures and images for: Validation of a cerebral hemodynamic model with personalized calibration in patients with aneurysmal subarachnoid hemorrhage
Source: Front Bioeng Biotechnol. 2022 Nov 25;10:1031600. doi: 10.3389/fbioe.2022.1031600 (PMC9732662; doi:10.3389/fbioe.2022.1031600)

## Supplementary

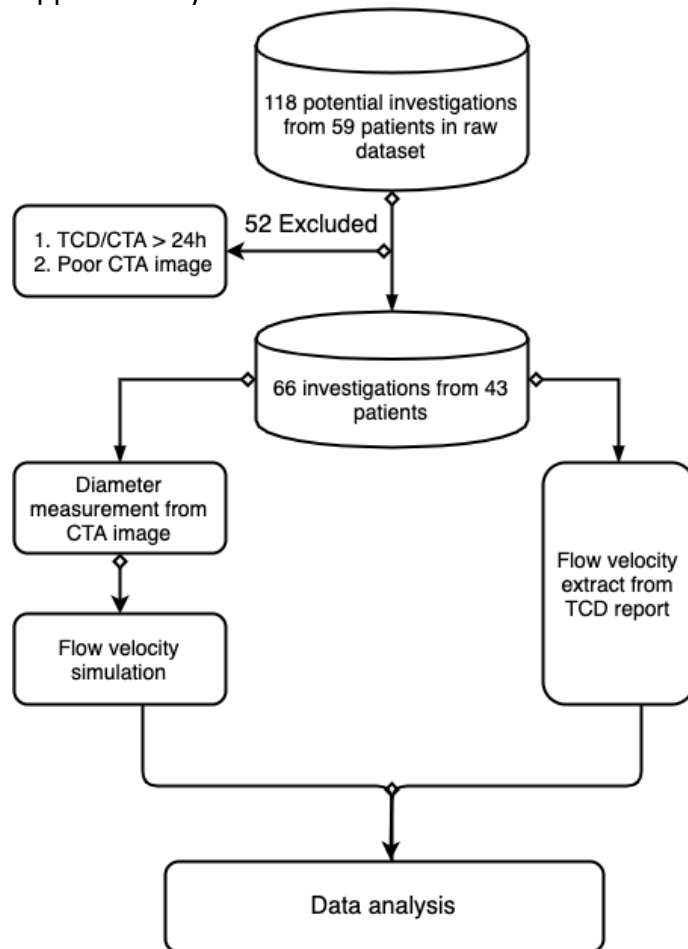

Study flow chat

Supplement: Supplementary file 3 [file Image1.PDF]
